# Long-term effects of systemic ceftiofur and ampicillin on the abundance and duration of shedding of resistant Gram-negative bacteria in the feces of healthy dairy cows: a randomized clinical trial

**DOI:** 10.3389/fvets.2026.1789173

**Published:** 2026-04-30

**Authors:** Juliano L. Gonçalves, Amanda T. F. Silva, Karla Vasco, Cara I. Robison, Shannon D. Manning, Lixin Zhang, Bo Norby, Rinosh Mani, Pamela L. Ruegg

**Affiliations:** 1 Department of Large Animal Clinical Sciences, College of Veterinary Medicine, Michigan State University, East Lansing, MI, United States; 2 Veterinary Diagnostic Laboratory, College of Veterinary Medicine, Michigan State University, Lansing, MI, United States; 3 Department of Veterinary Medicine, Federal Rural University of Pernambuco, Recife, Brazil; 4 Department of Microbiology, Genetics, and Immunology, College of Natural Science, Michigan State University, East Lansing, MI, United States; 5 Department of Animal Science, Michigan State University, East Lansing, MI, United States; 6 Department of Epidemiology and Biostatistics, Michigan State University, College of Human Medicine, East Lansing, MI, United States

**Keywords:** antimicrobial resistance, beta-lactams, dairy, fecal-resistant bacteria, treatment

## Abstract

**Introduction:**

We aimed to evaluate the long-term effects of systemic ceftiofur (CEF) and ampicillin (AMP) on the abundance and duration of fecal shedding of resistant Gram-negative (GN) bacteria from healthy dairy cows, as compared to cows that did not receive antibiotic treatment.

**Methods:**

Healthy lactating Holstein cows were randomly assigned to receive once daily treatments for 5 days with CEF (2.2 mg/kg subcutaneously, *n* = 8), AMP (11 mg/kg intramuscular, *n* = 8) or isotonic sodium chloride solution (25 cc, subcutaneously, *n* = 8). Fecal grab samples were collected before the initial treatment (day 0) and weekly for 11 weeks, beginning 2 days after the final treatment. Briefly, samples were diluted and inoculated in duplicate using a spiral plater on MacConkey agar or MacConkey agar supplemented with AMP or CEF. The phenotypic abundance of GN bacteria resistant to AMP or CEF was enumerated using a logarithmic scale and as a proportion of the total number of GN bacteria. Generalized linear mixed models for repeated measurements were used to compare the abundance of resistant bacteria among treatments over a 12-week clinical trial period.

**Results:**

The abundance of GN bacteria did not vary within or among treatments over the weeks of the trial. The abundance of GN bacteria resistant to AMP and CEF did not differ among treatment groups within a given week. After treatment, systemic treatment with AMP or CEF did not increase the proportion of resistant GN bacteria in feces of treated cows as compared to a control group that did not receive antibiotic treatment. Our preliminary molecular results suggest that the overall bacterial composition remained similar across groups. The relative abundance of antimicrobial resistance genes (ARG) revealed that the macrolide-lincosamide-streptogramin B (MLS), aminoglycoside, tetracycline, and elfamycin classes were the most predominant, whereas beta-lactams were moderate, ranging from 4 to 7%.

**Conclusion:**

In summary, a long-term effect of systemic treatment with AMP or CEF in healthy dairy cows produced minimal changes in the abundance of resistant GN bacteria, which indicates that systemically administered treatments of AMP and CEF had similar effects on the abundance and duration of resistant bacteria shedding in feces.

## 1 Introduction

Antibiotics are essential for combating bacterial diseases and improving human and animal welfare. Many of the same antimicrobial classes are used in both human and veterinary medicine, with organizations ranking their importance for treatment of bacterial diseases in humans (1). The term “critically important antimicrobials” (CIA) refers to antibiotic classes that represent the only compound (or one of a limited number) available to treat serious bacterial infections in humans. In the U.S., only 6 antimicrobials (ceftiofur, amoxicillin, ampicillin, oxytetracycline, penicillin, and narrowly restricted uses of sulfadimethoxine) are approved for systemic treatment of lactating dairy cows. Similarly, only 5 antimicrobials (amoxicillin, ceftiofur, cephapirin, hetacillin and penicillin) are labeled for intramammary treatment during lactation. In spite of being a CIA, ceftiofur (CEF) is a third-generation cephalosporin that is widely used in the U.S., due to favorable characteristics, such as lack of a milk withholding period (when systemically administered) and a flexible duration for the intramammary product. Systemic CEF is labeled for treatment of acute metritis, foot rot, and bovine respiratory disease while ampicillin (AMP), an aminopenicillin within the penicillin subclass of beta-lactams, is labeled for the treatment of bacterial infections caused by Gram-positive and Gram-negative (GN) bacteria, including bacterial pneumonia. Both are commonly used antimicrobials for treatment of metritis, but few studies have evaluated if use of systemically administered AMP impacts the abundance and dissemination of resistant GN bacteria differently than the use of CEF.

The large mass of antimicrobials used in animals may contribute to the emergence and dissemination of resistant bacteria, although the magnitude of risk is controversial (2–5). Antimicrobial use in food-producing animals accounts for an estimated 70–73% of global antibiotic sales. In the United States, the U.S. Food and Drug Administration (FDA) annually report sales and distribution of medically important antimicrobial drugs approved for use in food-producing animals. According to the 2024 FDA Summary Report, sales and distribution increased by 16% from 2023 to 2024, marking a departure from the relatively stable levels observed since 2017. The relationship between antibiotic use and its broader significance for dairy cattle production and public health remains unclear. However, if resistant bacteria are shed into the environment, they could potentially increase the risk of resistant infections in other cattle or individuals working in dairy farm environments. Amplification and dissemination of antimicrobial resistance (AMR) occurs in population-dense environments where antimicrobial use (AMU) is common, emphasizing that such practices can lead to the spreading of resistant bacteria among animals housed in close contact (6). A recent survey showed that >90% of consumers felt that AMU on dairy farms poses a threat to their personal health, and addressing these concerns is vital for maintaining trust in dairy products (7).

In this context, some countries have enacted voluntary or legislated reductions in the use of CIA in animal agriculture (8, 9). In the U.S., the wide availability of CEF and lack of a milk withholding period for systemic use make it a preferred choice for treatment of bacterial infections in dairy cows. While extra-label use of CEF in the U.S. is restricted, additional potential restrictions on the use of CEF in the U.S. are controversial because of concerns about limiting therapeutic options for treatment of food-producing animals (10). The potential emergence of resistance due to CEF use in dairy production has been explored in only a few studies, which diverge in their findings, perhaps due to differences in experimental design (i.e., sampling period), individual animal responses or sample size (11–13).

Understanding the relationship between administration of broad-spectrum antimicrobials and selection of AMR is an urgent need. In the U.S., few antimicrobials are approved to treat lactating dairy cows and AMP and CEF are the most commonly administered antimicrobials on dairy farms. While Gram-positive organisms may also harbor clinically relevant resistance determinants, the ecological and public health significance of beta-lactam resistance in enteric GN bacteria provided a focused and biologically justified framework for this investigation. We hypothesized that fecal shedding of bacteria resistant to AMP and CEF would increase after administration of therapeutic doses in otherwise healthy cows. We aimed to determine if systemic CEF and AMP administration affect abundance and duration of resistant GN bacteria found in the feces of healthy dairy cows. As an additional objective, we compared the abundance of GN-resistant bacteria and antimicrobial resistance genes (ARG) in cows that received treatment with AMP or CEF or remained untreated.

## 2 Materials and methods

### 2.1 Study design and treatments

This negatively controlled randomized complete block clinical trial was conducted from February to April 2023 at the Dairy Cattle Teaching and Research Center at Michigan State University, which contained approximately 230 lactating Holstein dairy cows. Mid-lactation healthy cows were eligible if they were not enrolled in other experiments, had not received antibiotic treatments during the last 90 d and had a current monthly SCC of ≤100,000 cells/mL. Twenty-four lactating cows housed in free stalls were blocked by parity and randomly assigned to receive daily treatments for 5 days with CEF hydrochloride (2.2 mg/kg subcutaneously, *n* = 8), AMP (11 mg/kg intramuscular, *n* = 8) or 0.9% sodium chloride solution injection (25 cc, subcutaneously, *n* = 8), using random numbers. The antibiotic dosage was defined as on the approved product label and was dosed based on each cow’s weight. Milk and meat withholding periods were defined by the product labels. Treatments were administered by the senior author and her laboratory manager (PLR, CR), and all other study personnel were blinded to treatment allocation until the initial analysis was complete. All cows were healthy at enrollment and throughout the study, and no morphological, physiological, or clinical changes in cows receiving antibiotics were observed compared with untreated animals.

Among the enrolled cows, fifteen multiparous dairy cows (averaging 43 kg of milk) were fed a high-concentrate protein diet, while nine primiparous cows (averaging 35.8 kg of milk) received a formulated diet for younger cows to meet their nutrient requirements based on their lower average milk yield. This study required FDA authorization for the treatment of healthy cows with antimicrobials, thus requiring us to minimize the number of cows used in the study. With 8 cows per treatment group, after adjusting for repeated sampling (using an intraclass correlation of 0.50), and a SD of 0.63 log10 with *α* = 0.05, the Bonferroni corrected power of this study to detect a 1-log difference in bacterial counts is 79%.

### 2.2 Sample collection, processing and abundance of phenotypic and genotypic antibiotic-resistant GN bacteria

Fecal grab samples were collected using clean obstetric sleeves, and samples were immediately transferred to sterile bags (Whirl-Pak^®^ Bags 3034-00) and cooled before transported to the Top Milk Laboratory at Michigan State University. Fecal samples were collected before the initial treatment (day 0) and then weekly for 11 weeks, beginning 2 days after the final treatment. Each sample was homogenized via hand massage of the bag, and 1 g of feces was aliquoted into a Falcon tube of 15 mL containing 9 mL of 1X phosphate-buffered saline for bacterial culture.

Media included MacConkey (MAC) lactose agar for GN bacteria and MAC supplemented with AMP or CEF. Amphotericin B was used in all plates to avoid fungal growth. Briefly, AMP (kept at 4 °C), CEF, and Amphotericin B (kept at -20 °C), were defrosted, weighed, diluted in sterile ultra-pure water or dimethyl sulfoxide according to the drug’s properties, vortexed, aliquoted, and stored as a stock solution at −20 °C. The final concentration was 32 μg/mL for AMP, 8 μg/mL for CEF and 4 μg/mL for Amphotericin B. Antibiotic concentrations were selected according to the minimum inhibitory concentration breakpoints published in reference materials (14, 15), and was based on values for Enterobacteriaceae. To ensure aseptic preparation of the plates, blank plates were incubated at 37 °C for 48 h under aerobic conditions prior to use. The quality control organisms for the non-supplemented MAC agar were *Staphylococcus aureus* ATCC 29,213 and *Enterococcus faecalis* ATCC 29,212. The antibiotic concentration on MAC that inhibited susceptible (S) bacteria and enabled the growth of resistant (R) strains was tested with the following control strains: *Escherichia coli* ATCC 25,922 (AMP-S, CEF-S), *E. coli* ATCC 35,218 (AMP-R, CEF-S), and three ESBL-producing *E. coli* strains (AMP-R, CEF-R) obtained from clinical samples in a prior study (15, 16).

Fecal samples were diluted to a concentration of 10^−1^ using a Falcon tube containing 1 g of feces and 9 mL of 1X phosphate-buffered saline, vortexed until homogenized, and the large particles were allowed to sit for 30 s. Subsequently, 50 μL was inoculated in duplicate on the non-supplemented MAC agar using a spiral plater (Spiral Biotech Autoplate 4000; Spiral Biotech, Inc., Norwood, MA) while 100 μL and 250 μL were inoculated in duplicate on supplemented MAC + AMP and MAC + CEF, respectively. Inoculated plates were incubated at 37 °C for 24 h under aerobic conditions.

The spiral plate quantification of bacteria was based on well-separated colonies in the outer region of the plate and was based on previous studies in our laboratory (16). Briefly, colonies were counted and divided by the volume of diluted fecal samples deposited in the segment regions of the plate. For the MAC and MAC + AMP plates, the count was divided by 5.5 or 11, corresponding to the volume in microliters, of the diluted fecal samples deposited in the segment regions for the 50 μL and 100 μL inoculated on the plates, respectively. The final enumeration was obtained by multiplying the total bacteria count by 10, (to correct for dilution of 10^−1^) and then by 1,000 for cfu/ml. For the MAC + CEF plates, the entire plate with inoculation of 250 μL of diluted fecal samples was counted and multiplied by 4 to obtain cfu/ml. Plates were counted in duplicate by 2 readers and were expressed as cfu/g feces.

Ninety-six out of 288 fecal samples were selected for DNA extraction based on a preliminary analysis of fluctuations in the shedding of GN-resistant bacteria (data not shown). Fecal samples were selected on day 0 (pre-treatment), 2 days after the final day of treatment (day 7, week 1), day 21 (week 4), and day 49 (week 8). Sequencing was performed on a NextSeq 1000 instrument (Illumina, Inc., San Diego, CA, United States). An equal mass of DNA (300 ng) from each sample was pooled per cow within each treatment group for the respective selected periods (days 0, 7, 21, and 49), resulting in 12 pools for shotgun sequencing. Further details on the steps of DNA extraction, library preparation, and sequencing, as well as the bioinformatic pipelines for bacterial diversity and antimicrobial resistance genes, are described in Supplementary File 1.

### 2.3 Statistical analysis

Analysis of variance for a completely randomized design of dairy cows assigned to each of treatments and its categorical variables was used to describe the population, assess the homogeneity of the groups, and identify potential biases or confounding factors that might influence the results.

The outcomes were the abundance and duration of shedding of resistant GN bacteria in the feces of healthy cows treated with CEF or AMP relative to the control (CTRL) group that did not receive antibiotic treatment. Abundance was measured either on a logarithmic scale (Log_10_(enumeration+1)) or as a proportion of the total GN bacterial count. The outcomes were compared among treatments over time, including the 12-week duration of GN bacteria shedding, using generalized linear mixed models for repeated measurements (PROC GLIMMIX, SAS software). Gaussian or negative binomial distributions were used to model the outcomes. The generalized linear mixed model was:

$$\begin{array}{l} \text{Outcome}\;\mathrm{ij}=L\mathrm{og}(\text{Area})+\beta 0+(\beta 1\times \text{Treatments})+\\ (\beta 2\times \text{Weeks}\_\text{of}\_\text{Sampling})+\\ (\beta 3\times \text{Stage}\_\text{of}\_\text{Lactation})+\\ (\beta 4\times \text{Parity})+(\beta 5\times \text{Plate}\_\text{Readers})+\\ u\mathrm{cow}i+v\text{week}j(\mathrm{cow}i)+\mathrm{\epsilon ij} \end{array}$$

where Log (Area) was the offset term to standardize (non-integer values) the final GN bacteria enumeration in cfu/g per plate. β_0_ was the intercept. β_1_, β_2_, β_3_ β_4_ and β_5_ were the regression coefficients for Treatments (CTRL, CEF and AMP), Weeks_of_Sampling (0 to 11), Stage_of_Lactation (early, ≤100 days; mid, >100 to ≤200 days, late, >200 days), Parity (1 and ≥2), and Plate_Readers (1 to 2). The *u*_cow*i*_ was the cow-specific random intercept *N* (0, *σ*^2^_cow_) and the *v*_week*j*(cow*i*)_ was the weeks of sampling within cow-specific random intercept *N* (0, *σ*^2^_week(cow)_). For all outcomes, parity, stage of lactation, diet, and plate readers were offered to the model as fixed effects. Backward selection was used to select variables that remained in the final models. The best models were selected based on convergence, and the model fit was based on lower values of 2 log-likelihood and a generalized chi-square/df ratio close to 1. The LSM was used for multiple comparisons after adjusting for Bonferroni to determine treatment effects and interactions over time. Differences were considered statistically significant at *p* < 0.05.

The direct outcome from sequencing was the normalized abundance of bacteria and ARG. The outcome of the read frequencies was used to determine the estimates of relative abundance. Descriptive relative abundance results for a selected group at genus level (*Escherichia, Salmonella, Campylobacter, Klebsiella, Proteus, Pseudomonas, Yersinia, Pasteurella, Mannheimia, Shigella, Bordetella, Moraxella, Aeromonas, Enterobacter, Morganella, Providencia* and *Serratia*), which have been previously reported in the context of bovine diseases or as carriers of ARGs (17), were summarized in the results section due to the high diversity observed among the 3,553 different genera identified (Supplementary File 2). We summed the read frequencies of ARG by classes of antimicrobials to determine the most frequently found (Supplementary File 3). Only antimicrobial classes with relative abundance >1% were reported with their respective ARG list by treatment and period (Supplementary File 4). Relative abundance estimates by treatment and time period for a selective group of GN at the genus level, along with the most frequent ARG grouped by class, were summarized using PROC MEANS (SAS software).

## 3 Results

### 3.1 Characteristics of the study population

All enrolled cows (*n* = 24) completed the study resulting in 288 fecal samples collected across the three-month sampling period. There were no differences among groups for days in milk (CTRL, 236 d SE 15; AMP, 230 d SE 11; CEP, 251 d SE 27; *p* = 0.79), parity (CTRL, 1.62 SE 0.18; AMP, 1.62 SE 0.18; CEP, 1.75 SE 0.16; *p* = 0.84), milk production (CTRL, 42 kg SE 2.7; AMP, 38.8 kg SE 3.4; CEP, 40.7 kg SE 2.4; *p* = 0.72), or SCC (CTRL, 4.39 log_10_SCC SE 0.12; AMP, 4.32 log_10_SCC SE 0.089; CEP, 4.42 log_10_SCC SE 0.096; *p* = 0.76). The proportions of cows that received high-concentrate feed (62.5%) or low-concentrate feed (37.5%) were equal among the three treatment groups. All cows remained healthy throughout the trial.

### 3.2 Phenotypic abundance of GN bacteria

Across all weeks, the abundance of GN bacteria did not vary among treatments (*p* = 0.98), with values of 9.77 log cfu SE 0.05 (AMP), 9.77 log cfu SE 0.05 (CEF), and 9.78 log cfu SE 0.05 (CTRL). A similar pattern was observed for GN bacteria resistant to AMP or CEF. Across all sampling weeks, no significant differences were found in the abundance of GN bacteria resistant to AMP among treatments (*p* = 0.40), with values of 3.48 log cfu SE 0.31 (AMP), 2.96 log cfu SE 0.31 (CEF), and 3.50 log cfu SE 0.30 (CTRL), nor for the abundance of GN bacteria resistant to CEF (*p* = 0.99), with values of −0.46 log cfu SE 0.21 (AMP), −0.42 log cfu SE 0.15 (CEF), and −0.44 log cfu SE 0.17 (CTRL).

**Figure 1:**
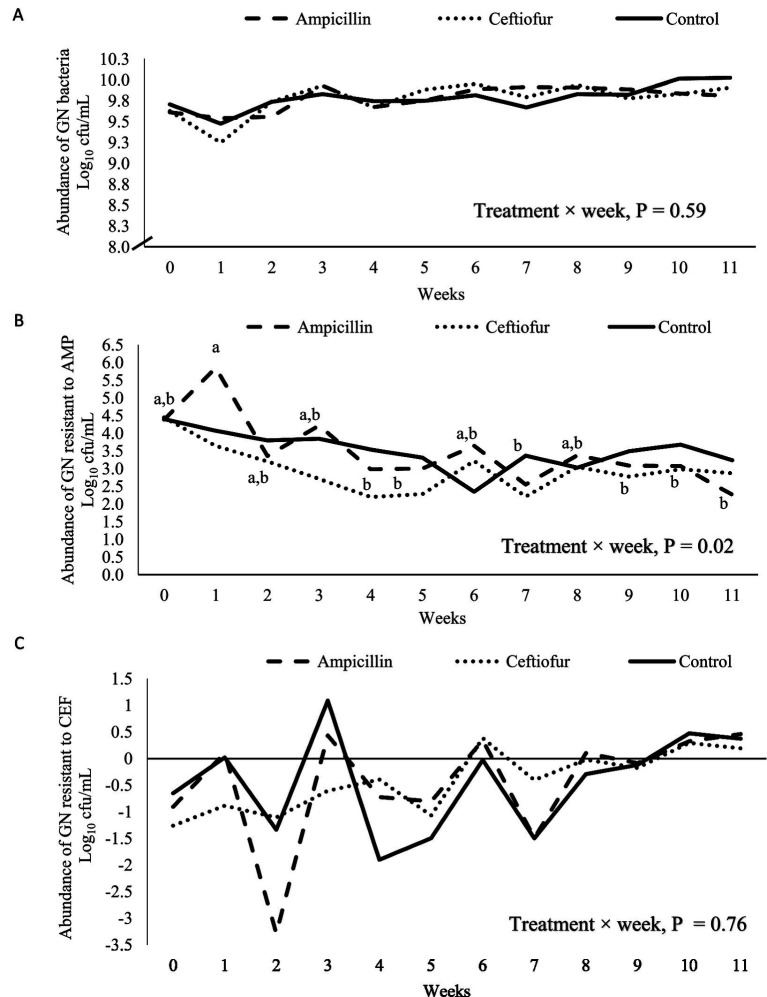
Abundance of Gram-negative (GN) bacteria in logarithmic scale **(A)**, with resistance to ampicillin (AMP) **(B)**, or ceftiofur (CEF) **(C)**, by week for mid-lactation healthy cows randomly assigned to control (*n* = 8), AMP (*n* = 8), or CEF (*n* = 8) treatment groups. Different letters within the graph indicate a statistical effect (*p* < 0.05).

The abundance of GN bacteria did not vary within or among treatments over the weeks of clinical trial (*p* = 0.59; Figure 1A). However, when we compared treatment groups over 11 weeks, there was a slight reduction in GN bacteria resistant to AMP during the AMP treatment course, with a decrease of 1.4 log cfu in week 4 to a level less than in the first week posttreatment (*p* = 0.02; Figure 1B). Subsequently, the abundance of GN bacteria resistant to AMP were slightly reduced following weeks, with a decrease of 2.1 log cfu in week 11 to a level less than in the first week posttreatment (*p* = 0.02; Figure 1B). Resistance to CEF fluctuated over the 11-week period, but no differences were observed within or among the treatment groups (*p* = 0.76; Figure 1C).

Across all weeks, the proportion of GN bacteria resistant to AMP did not differ among treatments (*p* = 0.54), with values of 30.9% ± 4.9 (AMP), 24.6% ± 4.9 (CEF), and 31.6% ± 4.9 (CTRL). Similarly, no differences were observed in the proportion of GN bacteria resistant to CEF (*p* = 0.55), with values of 9.5% ± 0.9 (AMP), 8.0% ± 0.9 (CEF), and 8.5% ± 0.9 (CTRL).

**Figure 2:**
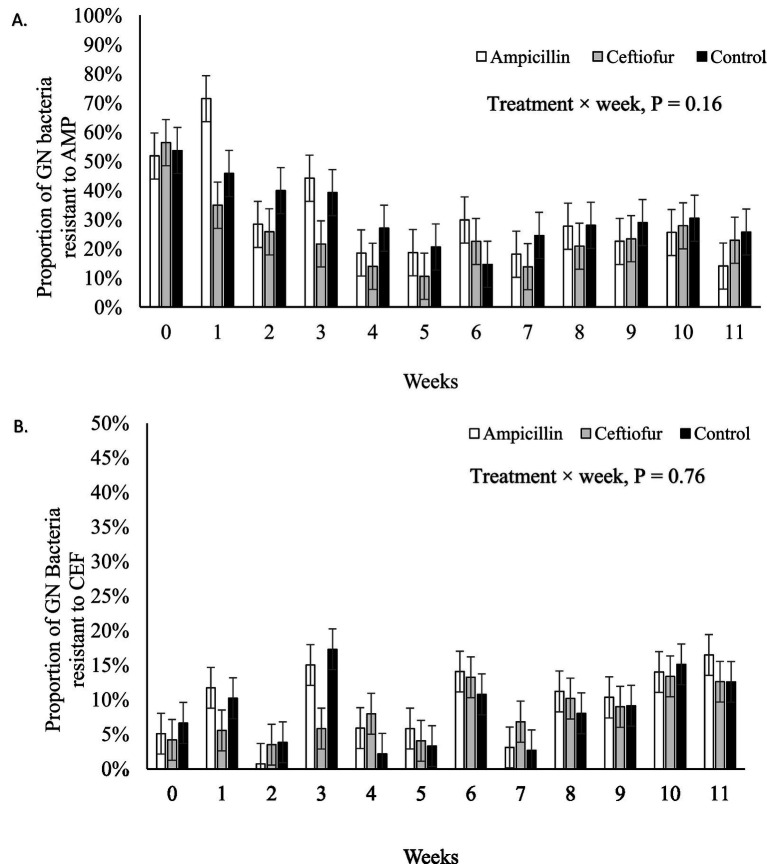
Proportion of Gram-negative (GN) bacteria with resistance to ampicillin (AMP) **(A)** or ceftiofur (CEF) **(B)** by week for mid-lactation healthy cows randomly assigned to control (*n* = 8), AMP (*n* = 8), or CEF (*n* = 8) treatment groups.

Systemic treatment with AMP or CEF did not increase the proportion of GN bacteria resistant to AMP (*p* = 0.16; Figure 2A) or CEF (*p* = 0.76; Figure 2B) in feces over 11 weeks when compared to a control group that did not receive antibiotic treatment. Interestingly, the abundance of GN bacteria resistant to AMP varied among cows within treatment (*p* < 0.01; data not shown). Before the administration of antimicrobials (d 0), one cow in each treatment group contributed > 50% of the bacteria that were resistant to AMP (CEF, 50.8% SE 8.9; AMP 57.3% SE 11.3; and CTRL 59.5% SE 12). Moreover, these 3 cows continued to shed bacteria resistant to AMP in the 11 following weeks. On the other hand, no variation in the proportion of GN bacteria with resistance to CEF was observed among cows within treatment over time (CTRL, *p* = 0.73; AMP, *p* = 0.42; and CEF, *p* = 0.10).

### 3.3 Relative abundance of GN bacteria and ARG

Rarefraction curves suggested that the sample size was large enough to estimate the relative abundance of bacteria and ARG at the class level. Supplementary File 2 provides the complete metagenomic taxonomic abundance, including not only Bacteria but also Archaea, Eukaryota, viruses, and unclassified sequences. In this study, which specifically targeted the bacterial domain, Clostridia was the most abundant bacterium at all treatments over the weeks out of 218 observed, accounting for about 48.7% (ranging from 42.5 to 51.5%) of all bacteria, followed by 20.3% of Bacteroidia (ranging from 18.2 to 23.7%), 5.9% of Spirochaetia (ranging from 3.7 to 10.4%), 3.9% of Bacilli (ranging from 3.6 to 4.1%) and 3.9% of Actinomycetes (ranging from 2.6 to 7.4%). These five classes accounted for over 80% of all bacteria (Supplementary File 5).

**Figure 3:**
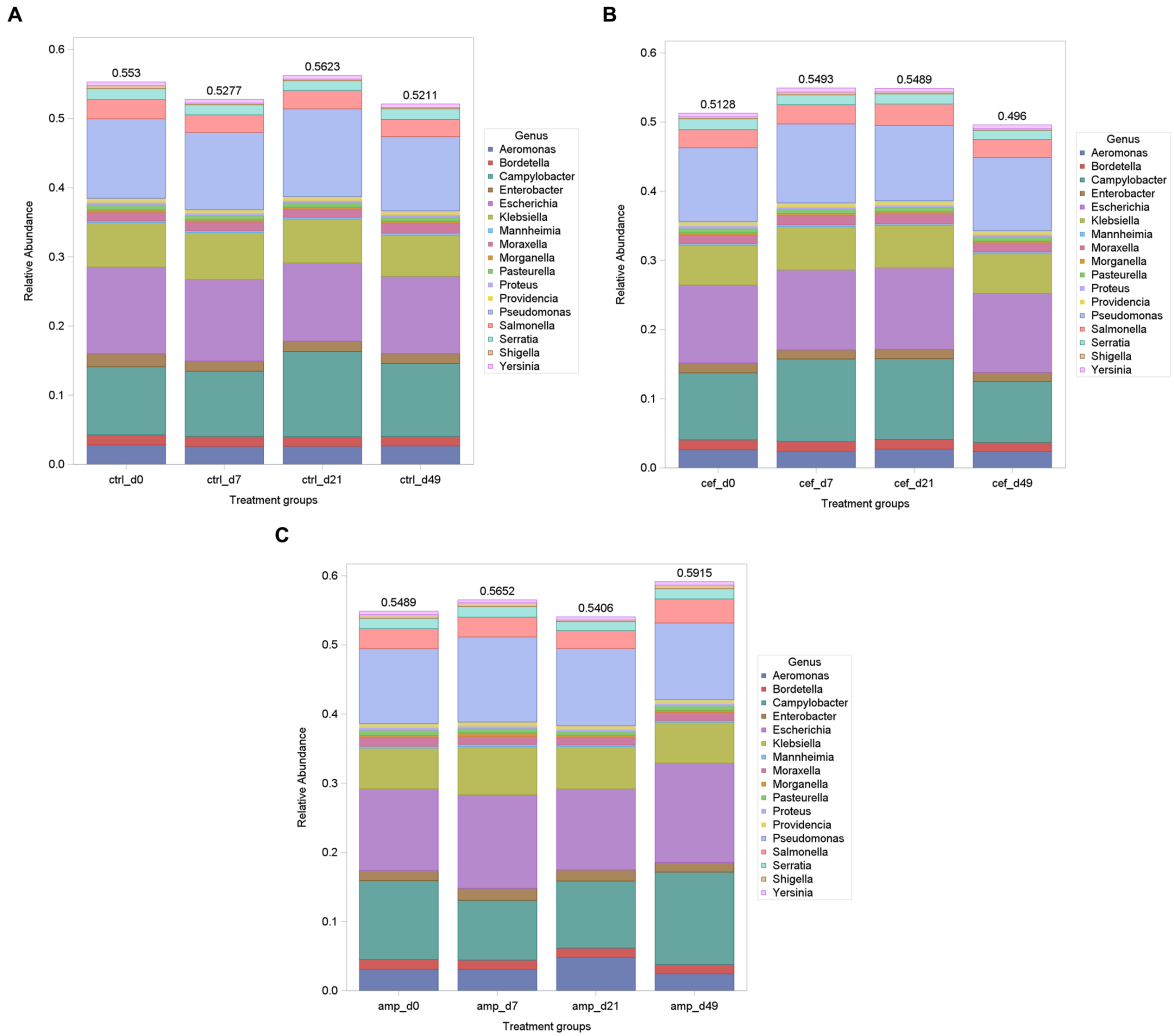
Relative abundance (%) of selected Gram-negative bacteria across treatments and sampling days. Each bar represents the cumulative relative abundance of selected 17 genera for a given treatment and timepoint (d0, d7, d21, d49). Treatments include **(A)** control (CTRL), **(B)** ceftiofur (CEF), and **(C)** ampicillin (AMP).

At the genus level, *Clostridium* was the first of top 10 most abundant bacterium at all treatments over the weeks, accounting for about 6.3% (ranging from 4.1 to 5.3%) of all bacteria, followed by 5.3% of *Treponema* (ranging from 3.2 to 9.8%), 4.5% of *Bacteroides* (ranging from 3.7 to 10.4%), 3.9% of *Pseudoflavonifractor* (ranging from 3.1 to 4.3%), 3.8% of *Prevotella* (ranging from 3.2 to 4.8%), 3.1% of *Alistipes* (ranging from 2.6 to 3.6%), 2.7% of *Ruminococcus* (ranging from 2.4 to 3.4%), 2.1% of *Bifidobacterium* (ranging from 0.9 to 5.7%), 1.6% of *Eubacterium* (ranging from 1.4 to 1.8%) and 1.3% of *Oscillibacter* (ranging from 1.0 to 1.5%). On average, 18% of the sequences could not be assigned to any bacterial genus. The selected group of GN bacteria ranged from 0.43 to 0.52% across all samples. Despite some variation, the overall composition remained similar across groups, with *Escherichia, Klebsiella,* and *Pseudomonas* being among the most prevalent genera (Figures 3A,B,C). However, the standard deviation of the relative abundance of *E. coli* during the days of AMP treatment (day 0: 0.12%, day 7: 0.14%; day 21: 0.12%; and day 49: 0.15%) appeared to be 6 and 2.2 times higher than that observed in the CEF and CTRL treatments, respectively. This may suggest that a lower and more consistent relative abundance of *E. coli* was observed during the CEF and CTRL treatments.

**Figure 4:**
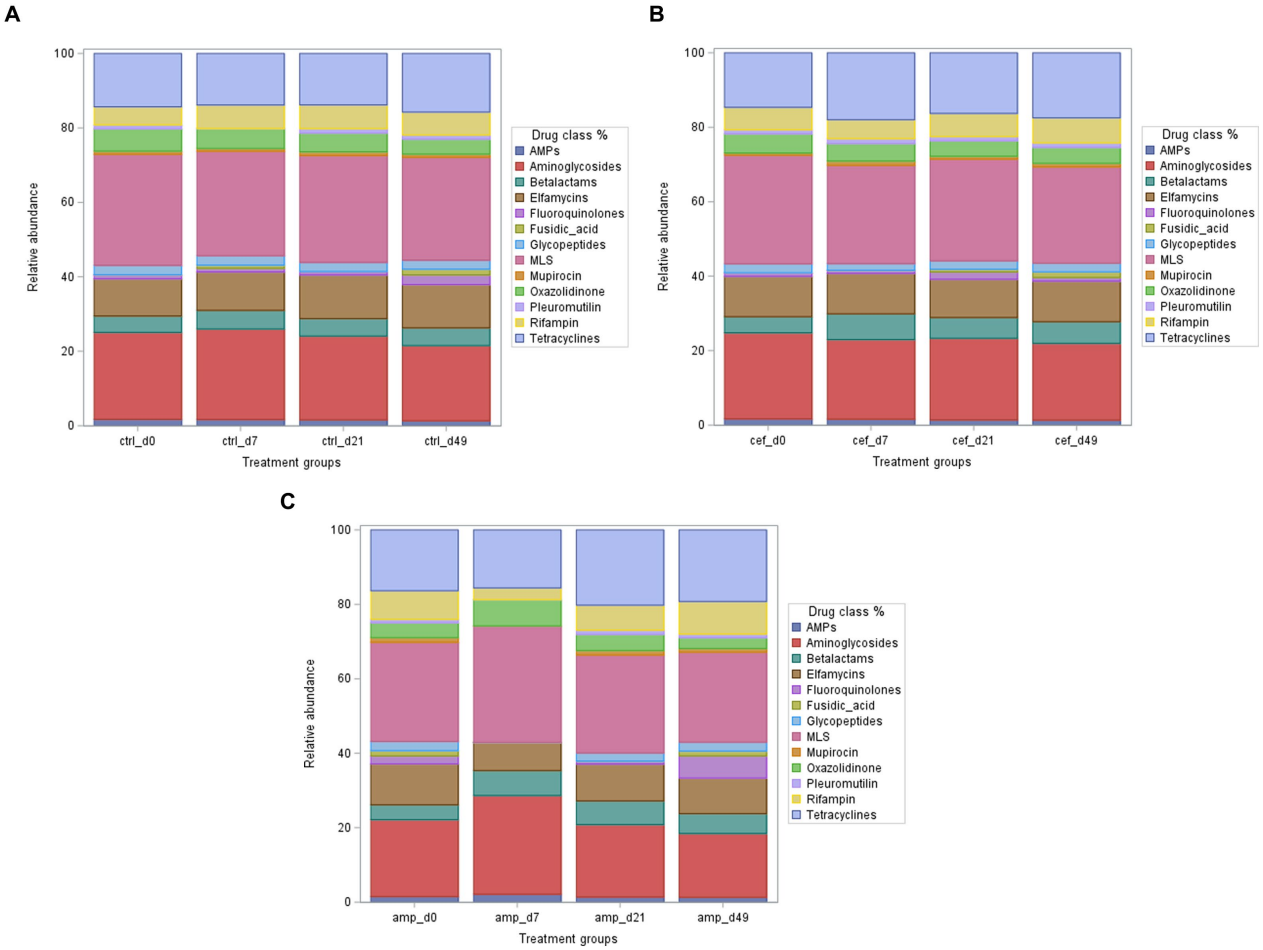
Relative abundance (%) of all antimicrobial resistance genes (ARG) summed by antimicrobial class across treatments and sampling days. Each bar represents the cumulative relative abundance of ARG for classes with >1% relative abundance at a given treatment and time point (d0, d7, d21, d49). Treatments include **(A)** control (CTRL), **(B)** ceftiofur (CEF), and **(C)** ampicillin (AMP), AMPs, antimicrobial peptides; MLS, macrolide-lincosamide-streptogramin B.

Our preliminary analysis of the relative abundance of ARG revealed that the macrolide-lincosamide-streptogramin B (MLS), aminoglycosides, tetracyclines, and elfamycins classes were the most predominant, with maximum values of 31.3, 17, 20, and 11%, respectively. In the beta-lactams, the group of particular interest for this study, the relative abundance of ARG was moderate, ranging from ~4–7% (Figures 4A,B,C). Resistance was mainly associated with *CFX* genes (likely conferring cefalosporin resistance), while regulatory or accessory genes, such as *ACI, ampCR,* and *MecI*, were present at very low levels (<1%; Supplementary File 4). Other classes, such as antimicrobial peptides (AMPs), Mupirocin, and Pleuromutilin, maintained low and stable abundance (~1–2%), indicating lower selective pressure. Glycopeptide resistance genes, including *vanR(C)*, were not detected in any treatment group, while the housekeeping gene *rpoC*, reported under the glycopeptide category, was detected across samples except in the AMP group on day 7.

## 4 Discussion

There are few antimicrobials approved for treatment of bacterial diseases in lactating dairy cows in the U.S. and AMP and CEF are most commonly used for systemic treatments (18, 19). Both antimicrobials are classified by the World Health Organization as CIA for human health but only CEF is classified as belonging to the highest priority categorization. While intramammary treatments for mastitis remain the primary contributor to AMU on dairy farms, systemic antimicrobials are frequently used to treat reproductive and respiratory diseases (20). Although a group of researchers did not find a significant link between intramammary antibiotic use and resistance, they did identify an association between systemic AMU and both increased resistance and multidrug resistance in isolated *E. coli* strains (21). A previous study identified a herd-level association between CEF use and the isolation of resistant organisms but found no such association at the individual cow level (11).

Given that disease treatment often necessitates higher antibiotic doses over short periods, understanding the long-term effect of therapeutic antibiotic dosages on resistance is crucial (22). Evidence of the short-period effect of CEF on fecal microbiota has already been investigated. A cohort study on dairy farms observed peak counts of Enterobacteriaceae resistant to CEF during treatment, with values returning to baseline shortly after treatment ended (23). A study by Fan and coworkers have found that colonic CEF concentrations initially increase and then drop to pre-treatment levels by day 8 (24). However, to our knowledge, studies investigating longer experimental periods have not been conducted. Assessing only short-term effects is insufficient without evaluating whether resistance persists for a significant period after treatment. Therefore, we aimed to evaluate whether the systemic administration of AMP or CEF influences the abundance or duration of resistant bacteria in feces of healthy dairy cows.

Extralabel treatments of healthy dairy cows with antimicrobials are not permitted by the FDA but was allowed for this trial. Use of healthy cows allowed contemporaneous treatments during the trial which minimized potential confounding based on parity, weather and dietary changes as well as the impact of disease itself. Furthermore, healthy cows may serve as a valuable model for assessing antimicrobial resistance pressure. They provide a controlled environment where the direct effects of antibiotics can be evaluated without interference from pre-existing diseases (25). This approach may minimize variability, making it easier to attribute observed effects directly to the antibiotic rather than to confounding factors such as infection severity, underlying health conditions, or immune responses. While healthy cows allowed us to compare outcomes during the same time period, our results should not be extrapolated to cows treated due to illness. Our findings indicate that systemic treatment with AMP or CEF in healthy cows produced only transient changes in the abundance of resistant GN bacteria, but further work is needed to determine if the same outcomes occur in cows treated for various diseases.

In our study, abundance of GN bacteria initially decreased, followed by an increase and stabilization, suggesting transient response to exposure to antimicrobials. The consistent abundance of GN bacteria among treatments aligned with previous findings, in which antibiotic-treated cows showed a significant initial decline in bacterial counts (16). This pattern mirrors that observed in our study, with only slight variations at different sampling times. Similarly, Taylor and coworkers observed this trend in *E. coli* counts following CEF treatment (26).

The proportion of GN bacteria resistant to CEF did not vary with time. Fluctuations in abundance of resistant bacteria in cattle have been attributed to the complex interplay between selection pressure and natural bacterial population dynamics. A cohort study on dairy farms found peak counts of Enterobacteriaceae with resistance to CEF during CEF treatment, but the values returned to baseline shortly after treatment ended (23). This result contrasts with the stable resistance of CEF observed in our CEF treatment group, which may indicate specific adaptation to a brief exposure to CEF during treatment or may be attributable to use of healthy (rather than ill) cows. Taylor and coworkers observed a high proportion of *E. coli* isolates resistant to third-generation cephalosporins in treated cows, with resistance levels returning to baseline posttreatment (24). Likewise, Vasco and colleagues reported an increase in the abundance of ceftiofur-resistant GN bacteria for up to 3 weeks after treatment initiation in the case group, but not in the healthy controls. However, by 4 weeks, the abundance had decreased to levels comparable to those of the control animals (27). A study by Boyer and Singer (22) suggested that the greater frequency of *E. coli* resistant to CEF in the CEF-treated group was due to a temporary reduction in the number of susceptible *E. coli*. Treatment with CEF led to an increase in bacterial sequences linked to resistance to beta-lactams and multidrug resistance, suggesting that the fecal excretion of resistance genes in dairy cows can be affected within a short timeframe of 3 days (28). These findings reported by Chambers and coworkers indicate that CEF exerts a broad, measurable, and immediate impact on the fecal metagenome of cows. On the other hand, in our study the proportion of GN bacteria with resistance to AMP showed more consistent reductions with time.

We found no differences in the proportion of GN bacteria with resistance to CEF or AMP among treatment groups within a given week, indicating a uniform resistance distribution. However, the temporal variation in resistance proportions highlights the dynamic nature of AMR in dairy cows, emphasizing the need for continuous monitoring. Overall, our results, regardless of the treatment group, showed that 29 and 8.7% of the 288 fecal samples contained GN bacteria resistant to AMP and CEF, respectively. This is consistent with previous findings (16), who reported greater resistance to AMP (90%) compared to CEF (24%), although they observed a greater overall proportion of bacteria resistant to AMP likely because they did not use the healthy cow’s approach employed in this study. Similarly, studies in Tennessee and Pennsylvania reported 21.2–30.8% of *E. coli* isolates with resistance to third-generation cephalosporin from dairy herds (29, 30). In contrast to our findings, Taylor and coworkers found that posttreatment, bacteria populations with resistance to third-generation cephalosporins in cows were unlikely to return to baseline levels, suggesting a competitive disadvantage after removing antibiotic pressure (26).

Similarly to the previous study (16), despite no differences between the abundance of resistant GN bacteria within the CEF and control treatment groups over the treatment time, variations in resistance were observed among individual cows within treatment. In Vasco and coworkers study a subset (25%) of CEF-treated cows shed higher levels of CEF-resistant bacteria for up to 2 weeks posttreatment compared with control cows (16). A somewhat similar pattern of persistent resistance was observed at the individual level, where one cow shed GN resistant to AMP at an average proportion exceeding 50% over 11 weeks. This uneven distribution suggests that individual animal effects may have influenced the observed abundance of AMP resistance and should be considered when interpreting treatment-level results.

Researchers have previously explored the descriptive and molecular epidemiology of antimicrobial-resistant GN enteric bacteria in the feces of healthy lactating dairy cattle (31). They isolated 42% of GN enteric bacteria resistant to tetracycline, 35% to AMP, 8% to florfenicol and 5% to spectinomycin from 213 lactating cattle studied. Among the *E. coli* isolates, resistance was found to be 93% for tetracycline, 78% for florfenicol, 48% for AMP, 20% for chloramphenicol, 18% for spectinomycin, and 11% for CEF. Fonseca and coworkers analyzed 1,086 fecal samples and reported that 24.5% of the *E. coli* isolates exhibited resistance to at least one antimicrobial (21). Notably, 20.7% of the isolates were resistant to tetracycline, whereas resistance to third-generation cephalosporins, fluoroquinolones, and carbapenems was observed in only 2.2, 1.4, and 0.1% of the isolates, respectively. Furthermore, 2.7% of the isolates were resistant to two antimicrobial classes, and 15% were resistant to three or more classes. In another study, dairy cows administered five doses of CEF at 2.2 mg/kg, exhibited a greater abundance of fecal *E. coli* isolates resistant to antibiotics on the fourth-, fifth-, and sixth days following treatment compared with untreated control cows (32). This period of posttreatment with CEF provided a window for the detection of ceftiofur-resistant *E. coli*, yet there was no clear evidence suggesting that the treatment induced the emergence or proliferation of these resistant strains (32). It is important to note that the mere identification of resistant isolates after antimicrobial treatment does not conclusively determine the selection pressure intensity or establish a direct causal relationship between antibiotic usage and the rise or expansion of resistance.

Genotypic AMR determinants were investigated using fecal pool samples rather than at the individual isolate level in our study. Although this may be considered a limitation, as it does not allow direct linkage between specific resistance genes and individual GN isolates, we conducted resistome analyses based on shotgun metagenomic sequencing data. This approach allowed us to evaluate the overall abundance of antimicrobial resistance genes present in the samples. Our preliminary molecular data showed that in the AMP treatment group, there was a peak in beta-lactam ARG on day 7 (6.7%), which would likely suggest a direct response to AMP administration, possibly due to the selection of bacteria carrying resistance genes for this class. Glycopeptide resistance genes, such as *VanR(C)*, were not detected in any of the treatment groups. The *rpoC* gene reported under the glycopeptide category is a housekeeping gene that may act as an antibiotic target in mutation-based resistance mechanisms rather than representing an acquired resistance gene. Core housekeeping genes were detected because the reference database includes target genes associated with resistance via point mutations. In the initial output, total gene abundance was reported without distinguishing between wild-type and resistance-conferring variants. The transient absence of *rpoC* in the AMP group on day 7 may reflect a short-term shift in the relative abundance of bacteria carrying this target gene following AMP treatment. Its reappearance at later time points (on days 21 and 49) likely reflects recovery of the microbial community rather than changes in acquired glycopeptide resistance. In the CEF treatment group, values remained relatively stable over time, which might indicate that CEF exposure did not lead to major changes in the relative abundance of beta-lactam genes during the evaluated periods. In Pennsylvania dairy herds, a higher incidence of resistance was observed in ESBL-producing *E. coli* isolates (29). It is likely that this increased resistance they observed is consistent with previous findings suggesting that ESBL isolates often harbor antimicrobial resistance genes that confer co-resistance to antimicrobial classes beyond beta-lactams (21, 30).

Although there were only 24 cows enrolled in this study, the differences in abundance of resistant GN bacteria across time were very small, indicating that many cows would need to be enrolled to a relatively small effect of treatment. Our findings are, however, preliminary and should be confirmed with larger studies conducted in cows treated for disease.

Our study provides important information relative to comparing abundance of resistant GN bacteria in healthy dairy cows receiving the most commonly administered antimicrobial treatments. Our study was designed to include only healthy cows that met specific inclusion criteria, with the intention of minimizing confounding based on differences in diets, environment and parity. As such, our conclusions apply only to healthy cows and further studies are needed. In addition, because the animals were housed at a university-managed dairy farm where antimicrobial use is generally prudent and controlled, cumulative antimicrobial exposure may have been lower than that of cows managed under different commercial conditions. Consequently, the fecal microbiota of these animals may respond differently to antimicrobial administration and may recover more rapidly than microbiota of cows with greater historical antimicrobial exposure.

## 5 Conclusion

A long-term effect of systemic treatment with AMP or CEF in healthy dairy cows produced minimal changes in the abundance of resistant GN bacteria. Variation in resistance was observed among individual cows. Our preliminary findings indicate that systemically administered treatments of AMP and CEF had similar effects on the abundance and duration of resistant bacteria shedding in feces.

## Data Availability

The original contributions presented in the study are publicly available. This data can be found here: NCBI repository, BioProject ID: PRJNA1403569.
