# Supplementary material for: Long-term effects of systemic ceftiofur and ampicillin on the abundance and duration of shedding of resistant Gram-negative bacteria in the feces of healthy dairy cows: a randomized clinical trial
Source: Front Vet Sci. 2026 Apr 30;13:1789173. doi: 10.3389/fvets.2026.1789173 (PMC13174580; doi:10.3389/fvets.2026.1789173)
Supplement: Supplementary file 1 [file supplementary_file_1.docx]

|  | A. |  |
| --- | --- | --- |
|  | B. |  |
|  | C. |  |
| **Figure 1.** Abundance of Gram-negative (GN) bacteria in logarithmic scale (A), with resistance to ampicillin (AMP) (B), or ceftiofur (CEF) (C), by week for mid-lactation healthy cows randomly assigned to control (n = 8), AMP (n = 8) or CEF (n =8) treatment groups. Different letters within the graph indicate a statistical effect (P < 0.05). | | |

|  |  |  |
| --- | --- | --- |
|  |  |  |
| **Figure 2.** Proportion of Gram-negative (GN) bacteria with resistance to ampicillin (AMP) (A), or ceftiofur (CEF) (B), by week for mid-lactation healthy cows randomly assigned to control (n = 8), AMP (n = 8) or CEF (n =8) treatment groups. | | |
